# *Health Care Science*—Why another journal and why we will be different?

**DOI:** 10.1002/hcs2.2

**Published:** 2022-07-07

**Authors:** Zongjiu Zhang, Jiefu Huang, Wong Tien Yin, Haibo Wang

**Affiliations:** ^1^ Institute for Hospital Management of Tsinghua University Beijing China; ^2^ China National Organ Donation and Transplantation Committee Beijing China; ^3^ Tsinghua Medicine, Tsinghua University Beijing China; ^4^ The First Affiliated Hospital of Sun Yat-Sen University Guangzhou China

**Abbreviations**
CrediT: Contribution Roles Taxonomy
DORA: Declaration on Research Assessment
ESCI: The Emerging Sources Citation Index
HCS: Health Care Science
NCDs: noncommunicable diseases
SCIE: The Science Citation Index Expanded

Why another journal? As of 2021, there are over 7000 SCIE (The Science Citation Index Expanded) or ESCI (The Emerging Sources Citation Index) indexed journals listed under the category of Clinical Medicine in Clarivate Journal Citation Reports, of which 263 journals are featuring health care sciences, policy, and services. How would another journal help? What gaps are we trying to fill?

First, the world is undergoing significant transformative changes not seen for a whole century. For example, the COVID‐19 pandemic has ravaged healthcare systems and societies for 3 years now from the beginning of 2020. The pre‐COVID global healthcare agendas have been driven off the tracks by the persistent substantial disruption of health services worldwide during the pandemic [1]. The target of United Nations Sustainable Development Goal 3.4 (by 2030, reduce by one‐third premature mortality from noncommunicable diseases, NCDs) is now at the risk of a long‐term upsurge in deaths from NCDs [2]. The once‐in‐a‐century global health crisis has provoked us to reflect deeply on the need for a faster and larger systematic change in healthcare systems.

In the past decades, a large amount of new biomedical research has pushed forward the frontiers of medicine, allowing the discovery of new therapies and treatments, yet the percentage of publications on health policy and service research remained under 15% in top international medical journals. There is a huge gap between the current body of research and the need for scientific evidence on effective and efficient health care delivery systems. To bridge this gap, we established *Health Care Science (HCS)*. HCS will not be another journal that focuses on basic or clinical medical research and compete with so many others. Instead, *HCS* will be moving from patients and diseases to health services and systems, intending to create an open platform that attracts professionals from various disciplines to exchange concepts, methods, and scientific findings in healthcare service research. *HCS* will be particularly interested in health systems innovations, not only specifically in health service research but also in the impact and challenges on the healthcare system arising from the fast‐pacing scientific and technological advances in clinical practice. The breakthroughs in medical technology and clinical research are exciting but never the panacea. If not properly regulated and implemented, the associated risks could outweigh the benefits. There is an urgent need for scientific and systematic thinking in health service delivery and medical application management.

**Table 1:** Significant challenges in the current range of journals and solutions proposed by Health Care Science

Challenges (Harlan Krumbolz, 2015)		How HCS will address these challenges
**Too slow**	Long lag throughout manuscript formatting, submission, (rejection), review, revision, and acceptance.	1. HCS adopts “free format” submission 2. HCS welcomes preprints and actively seeks for submission from preprint servers. 3. HCS welcomes referred manuscript not suitable for other journals (accompanied by previous peer‐reviews).
**Too expensive**	Increased cost for publication and open‐access option.	1. HCS offers waivers and discounts to authors in low‐ and middle‐income countries. 2. HCS waives the Article Publication Charge for manuscripts submitted from 2022 to 2024. 3. HCS is within Wiley Open Access program which partners with agencies or institutions to support the publication charge.
**Too limited**	Strict constraint on article length and contents that limited the readers from judging the quality or reproducing the findings.	1. HCS will adopt “free format” submission of articles. 2. HCS will be flexible and provide broad word limits as suggestions only, and allow comprehensive supplementary materials where necessary.
**Too powerful** **Too unreliable**	1. Too much power from journal editors on decision‐making. 2. Inconsistent and non‐transparent peer‐review and decision process.	1. HCS will declare conflicts of interest that Editors might have, and publish how decision‐making process was handled. 2. To safeguard research integrity, which is the core of any academic journal, HCS adopts the Contribution Roles Taxonomy (CrediT) to make sure all authors' individual contributions are correctly recognized. In addition, HCS expects data sharing, where all research articles will be published accompanied by clear data availability statement.
**Wrong metrics**	Disproportionate attention to paper citation and journal impact factor.	HCS under Wiley will adopt the San Francisco Declaration on Research Assessment (DORA) to shift emphasis away from journal‐based metrics and assessment, toward article‐level metrics and individual author contribution.
**Too parochial**	Lack of diversity in editorial team makeup.	HCS has invited a diverse international editorial board, with a younger “emerging” editorial board. HCS will also strive to bring in opinions and experiences from authors of diverse background, including developed and low‐ and middle‐income countries.

Second, there are significant challenges in the current range of journals (Table 1). We HCS proposes real solutions. The real‐world application of healthcare innovations does not allow much time for waiting. It is critical that the experience in healthcare management and service delivery be shared in a rapid and timely manner. And that is why we are striving to make changes to the current journal publishing norm. (1) *Speed*—To reduce the lag throughout manuscript formatting, submission, (possible rejection), peer‐review, revision, and acceptance, *HCS* adopts “free format submission,” actively seeks submission from preprint servers, and welcomes transferred manuscripts not suitable for other in‐network journals, accompanied by previous peer‐reviews. (2) *Transparency*—To ensure a more consistent and transparent peer‐review and decision process, *HCS* requires the declaration of conflicts of interest that editors and journals might have and will publish decision‐making process was fairly handled. (3) *Metrics*—To put emphasis on practical healthcare delivering, *HCS* is keen to shift away from disproportionate attention to journal‐based metrics and assessment (e.g., impact factor). We stand with our publisher, Wiley, who recently endorsed the San Francisco Declaration on Research Assessment (DORA) to shift emphasis toward article‐level metrics and individual author contribution [3]. (4) *Diversity*—To diversify the editorial team, *HCS* has composed an international editorial board, a vibrant emerging editorial board, as well as a strong advisory board to get inputs and insights from professionals of various backgrounds. We are also striving to bring in opinions and experiences from authors of diverse geographic regions, covering developed and low‐ and middle‐income countries. (5) *Integrity*—To safeguard research integrity, which is the core of any academic journal, *HCS* adopts the Contribution Roles Taxonomy (CrediT) to make sure all authors' individual contributions are correctly recognized. In addition, *Health Care Science* expects data sharing, where all research articles will be published accompanied by a clear data availability statement [4].

In conclusion, *HCS* is aimed to connect and integrate the academic and the administrative sectors, to bring the world new perspectives on medical innovations and healthcare systems, through the lenses of legislation, policy, ethics, and humanity. The world is still at a critical conjecture of the COVID‐19 pandemic. At this moment, the proverb from the ancient Roman philosopher Seneca seems even more meaningful: “We are the waves of the same sea.” We hope that *HCS* will serve as a driving force to bring together the world's first‐class scientific research on health service research, contributing to the global community of health and helping in shaping a brighter future for all mankind.


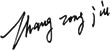


**Editor‐in‐Chief**

**Zongjiu Zhang, MD**


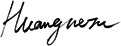


**Honorary Editor‐in‐Chief**

**Jiefu Huang, MD**


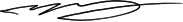


**Honorary Editor‐in‐Chief**

**Wong Tien Yin, MBBS, PhD, MPH**


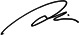


**Executive Editor‐in‐Chief**

**Haibo Wang, MBBS, MSc, MPH**

## AUTHOR CONTRIBUTIONS

**Zongjiu Zhang**: conceptualization (equal); writing original draft (equal); review (equal). **Jiefu Huang**: review (equal); editing (supporting). **Wong Tien Yin**: conceptualization (equal); review (equal); editing (supporting). **Haibo Wang**: conceptualization (supporting); writing original draft (equal); review (equal); editing (lead).
